# Movement Networks of Domestic Livestock Susceptible to Food and Mouth Disease in Thailand

**DOI:** 10.1155/vmi/6680248

**Published:** 2025-09-11

**Authors:** Sukanya Thongratsakul, Nattachai Warrasuth, Chaithep Poolkhet

**Affiliations:** ^1^Akkhraratchakumari Veterinary College, Walailak University, Tha Sala, Nakhon Si Thammarat, Thailand; ^2^Department of Livestock Development, Ministry of Agriculture and Cooperatives, Bangkok, Thailand; ^3^Faculty of Veterinary Medicine, Kasetsart University, Kamphaeng Saen, Nakhon Pathom, Thailand

**Keywords:** exponential random graph models (ERGMs), foot and mouth disease (FMD), movement networks, social network analysis, Thailand

## Abstract

Foot and mouth disease (FMD) is one of the most common diseases in livestock that causes great losses to industrial livestock production. FMD may spread through animal movements. Therefore, this study aimed to analyze the patterns of domestic livestock movements susceptible to FMD using social network analysis and exponential random graph models (ERGMs). Three years of secondary data on animal movements from January 2017 to December 2019 were analyzed and simulated. We found that pig movements account for the largest proportion of FMD each year. Central Thailand was seemingly prominent for animal movements. For ERGMs, the microstructures: edges, mutual, TwoPath, and triangle, were tested and appeared in the movement networks for all the years. The results of our data analysis would help relevant authorities better understand animal movement patterns resulting in superior planning and surveillance for disease control. Moreover, this study's findings can be applied to analyze animal movement patterns globally, aiding in the development of effective disease prevention and control policies. Understanding these patterns enhances planning and surveillance for disease control across various regions.

## 1. Introduction

Foot and mouth disease (FMD), one of the most common diseases in livestock, causes great losses in industrial livestock production. A previous study estimated the global annual impact of FMD from visible production losses and vaccination to range from US$ 6.5 to 21 billion [1]. FMD is caused by the FMD virus (FMDV), from the family Picornaviridae, genus Aphthovirus. FMDV was classified into seven serotypes: A, O, C, SAT1, SAT2, SAT3, and Asia1. Domesticated animals, such as cattle, pigs, sheep, goats, and buffalo, are susceptible to FMDV. Other animals, such as wild cloven-hoofed species, are less susceptible to FMDV compared to domesticated cloven-hoofed species [2]. In Thailand, only Serotypes A, O, and Asia1 were reported. From 2017 to 2021, 43–178 outbreaks commonly occurred in cattle, while the occurrence of FMD was reported in buffaloes, pigs, goats, and sheep in a few outbreaks. The infected animals were identified as Serotypes A and O [3].

Animal movement is a key behavior that plays an important role in spreading infectious diseases [4]. Particularly, the movement of infected and asymptomatic animals influences the spread of the disease. A ban on animal movement was imposed after disease detection was deemed necessary to prevent the spread of FMD after learning from FMD epidemics in the United Kingdom [5]. In Cameroon, the researchers demonstrate evidence of the involvement of transboundary trade cattle in FMD transmission [6]. Likewise, a previous study suggested the limited animal movement in Sudan to be one of the key control measures for reducing FMD spreads [7].

Social network analysis (SNA) is one of the best tools for evaluating the relationship between animal movement and disease spread. For the FMD epidemic in the United Kingdom, animal movement was associated with the actor, contact of the animal, and disease occurrence. The centrality value of the SNA, such as betweenness, can be used to identify the key actor for epidemics [8]. A previous study reported that the actor with a high outdegree was associated with the epidemic size [9]. A study in South Africa presented that the most influential actors for FMD spreads were actors with high values of outdegree and outcloseness [10]. Increasing network relationships reportedly results in network complexity. Network modeling, such as exponential random graph models (ERGMs), can offer a better understanding of the networks. Thus, analyzing data with ERGMs would help relevant authorities implement the most effective control measures for FMD. The ERGM is a probabilistic model of ties among actors based on empirical data [11, 12]. This model would aid in constructing tie formation and has been used to study predicted network behaviors [11, 13].

This study aimed to analyze the patterns of domestic livestock movements susceptible to FMD using SNA and ERGMs. The results of this study can be applied in the control and surveillance of disease.

## 2. Methods

### 2.1. Study Design and Data Collection

Three years of secondary data on animal movements from January 2017 to December 2019 were obtained from a database named “e-Movement” of the DLD in Thailand. The data in each calendar year were used to represent annual movement networks. The movement data belonged to live domestic livestock, such as cattle, buffalo, pigs, goats, and sheep, susceptible to FMD. The level of analysis was evaluated in epidemiological units at the provincial level. All the provinces of Thailand (*n* = 77) were included in this study. The movement data were defined as the origin of movement, the destination of movement, the identification number, the date, and the animal species. In this study, we assumed all animal species to be equally susceptible to FMD for analytical purposes because the available movement dataset did not include species-specific FMDV serotype information. We acknowledge that in reality, susceptibility varies across species; for example, cattle are highly susceptible to all serotypes, while pigs serve as amplifying hosts for Serotypes O and Asia-1.

For ethical issues, all the procedures in this study were checked and followed the Declaration of Helsinki. However, this study used secondary data, which do not contain any studies with human or animal participants. The DLD approved all the received data for conducting anonymous research.

### 2.2. Network Properties and Data Manipulation

Directed, valued, and asymmetric networks were manipulated for data analysis from the annual networks (Supporting Data 1, 2, and 3). The data of the origin of movement and destination of the movement were created as edge list data in Microsoft Excel (Microsoft, Redmond, Washington, USA). Thereafter, the edge list data were converted to matrix data using UCINET release 6.742 (Analytic Technologies, Lexington, Kentucky, USA). Thus, we collected three sets of UCINET files for data analyzed for descriptive networks and network hypothesis testing. For network modeling, UCINET files were converted to .net files using UCINET release 6.742. Therefore, the data were simulated for ERGMs using R release 4.1.3 with “statnetWeb” packages [8, 14]. This tool enables users to import network files, define model terms, perform MCMC estimation, and evaluate goodness-of-fit (GOF), all without the need for coding. As a GUI-based approach, it does not produce R scripts.

### 2.3. Data Analysis and Visualization

#### 2.3.1. Descriptive Network Analysis and Hypothesis Testing of Networks

Descriptive network analysis was used to descript the individual actor (province) importance for three networks. Outdegree, indegree, outcloseness, incloseness, and betweenness (Table 1) were measured using the raw scores and normalized values [15, 16]. For network hypothesis testing, the matrix data of animal movements were computed using QAP correlation for network similarity [16]. By default of UCINET, 5000 permutations were performed, and the coefficient and probability values were reported.

#### 2.3.2. Network Modeling

ERGMs were used to describe the microstructure for directed, valued, and asymmetric networks. Edges, mutual, TwoPath, and triangle (Table 1) were tested using R with “statnetWeb” packages [8]. The selected microstructures for ERGMs were based on data compatibility, model degeneracy and stability, and the computerized system (we used a personal computer that contained 128 gigabytes of computer memory and a 2x Intel Xeon SILVER 4114 Processor). The GOF was used to ascertain the quality of the overall fit of the models. This GOF was based on 100 simulated networks from the empirical data.

#### 2.3.3. Mapping

All the movement data were plotted and visualized using ArcGIS release 10.8.2 (ESRI, Redlands, California, USA). The coordinates of the provinces (Supporting Data 4) were collected from Google Sheets (Google LLC, Mountain View, California, USA) using Geocode function. The kernel density and linear directional mean were also computed in each network. Moreover, the results of individual actors in each network were presented on the map of Thailand using choropleth maps; quantile classification was used for this presentation and assigned into five classes.

## 3. Results

### 3.1. General Information

Based on the movement data from 2017 to 2019, the total number of movements was 417,147, 451,265, and 527,214 times for 2017, 2018, and 2019, respectively. In 2017, the number of movements for pig, cattle, buffalo, goat, and sheep was 315,304 (315,304/417,147; 75.58%), 74,445 (74,445/417,147; 17.85%), 22,926 (22,926/417,147; 5.50%), 3804 (3804/417,147; 0.91%), and 668 (668/417,147; 0.16%) times, respectively. In 2018, the number of movements for pig, cattle, buffalo, goat, and sheep was 341,396 (341,396/451,265; 75.65%), 80,394 (80,394/451,265; 17.82%), 25,000 (25,000/451,265; 5.54%), 3872 (3872/451,265; 0.86%), and 603 (603/451,265; 0.13%) times, respectively. In 2019, the number of movements for pig, cattle, buffalo, goat, and sheep was 403,245 (403,245/527,214; 76.49%), 94,469 (94,469/527,214; 17.92%), 25,172 (25,172/527,214; 4.77%), 3891 (3891/527,214; 0.74%), and 437 (437/527,214; 0.08%) times, respectively.

### 3.2. Descriptive Network Analysis

The number of actors for each year was 77, while the number of ties was 3546, 2871, and 2971 for 2017, 2018, and 2019, respectively. We found that the mean of the raw scores of all the centrality values was high every year, resulting in a high mean of normalized values (Table 2). In each year, the mean of each centrality value showed close similarity in the same parameter with the other years. For the variation of the centrality values, the outdegree and indegree had high standard deviation (SD) when considered together with its mean. Moreover, a permutation test for the correlation coefficient demonstrated that strong significant correlation among all the pairs of the annual movement network (*r* = 0.930–0.979; *p* < 0.01) (Table 3).

**Figure 1:**
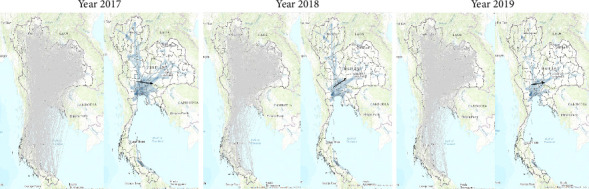
The map of Thailand represents the movement of domestic livestock susceptible to foot and mouth disease (FMD) between the years 2017 and 2019. For each year, the left figure shows all the paths of movement using geodesic distance, and the right figure shows the kernel density with a linear directional mean (black arrow), which was manipulated from the left figure. Source: Map generated by ArcGIS 10.8.2 software.

Figure 1 shows that the movement pattern of susceptible domestic livestock to FMD had a nearly similar appearance for all 3 years. Based on the results of kernel density (Figure 1; right figure of each year), the movements were dense in central Thailand that connected to the lower parts of the northeastern and central part of northern Thailand. Based on the geodesic path, the linear directional mean of all networks had a west to east direction in the central part of Thailand.

**Figure 2:**
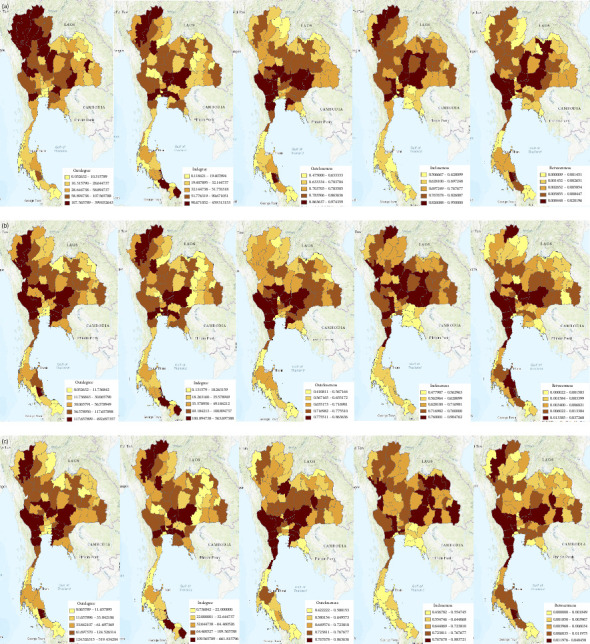
The map of Thailand represents the provincial centrality values by years. The first to third row represents the years 2017 (a), 2018 (b), and 2019 (c), respectively. The first to fifth column shows the normalized outdegree, normalized indegree, normalized outcloseness, normalized incloseness, and normalized betweenness, respectively. The color ramp in each figure represents the normalized centrality value from the smallest (yellow) to the largest (dark brown). Source: Map generated by ArcGIS 10.8.2 software.

Figure 2 shows that the outdegree value was prominent in some provinces in the northern, western, central, eastern, and lower parts of northeastern Thailand for all 3 years. However, each year, the number of provinces with the highest outdegree value could be slightly different (first column of Figure 2). For indegree, the value was prominent in some provinces in northern, central, eastern, and lower parts of southern Thailand for all 3 years (second column of Figure 2). For the outcloseness value (third column of Figure 2), some provinces located in the western, central, eastern, and lower parts of northeastern Thailand were prominent. For the incloseness value (fourth column of Figure 2), the prominent provinces were scattered. It was demonstrated that in 2017, some northern, western, central, and northeastern Thailand provinces were prominent. In 2018, the provinces in the northern, western, northeastern, and upper parts of southern Thailand were prominent. In 2019, the western, central, and northeastern Thailand provinces were prominent. For betweenness (fifth column of Figure 2), in 2017, the value was prominent in some provinces in the western, central, northeastern, and lower parts of southern Thailand. In 2018, the provinces in the northern, western, central, lower part of northeastern, and lower part of southern Thailand were prominent. In 2019, some provinces in the northern, western, central, northeastern, and lower parts of southern Thailand were prominent. In conclusion, when considering the overall parameter for all three years, the area corresponding to central Thailand was most prominent for the movement of susceptible domestic livestock from 2017 to 2019.

### 3.3. Network Modeling

Results of the ERGMs demonstrated most microstructures to be statistically significant (*p* < 0.01) (Table 4). Only edges in 2018 and 2019 did not show statistical significance. Interestingly, higher order microstructures, such as TwoPath and triangle, appeared more than edges and mutual in every year of the movements. In addition, the GOF indicated that all the microstructures from the sample data had a good model fit.

## 4. Discussion

This study evaluated the movement networks of susceptible domestic livestock possibly associated with FMD spreads in Thailand using SNA and ERGMs. We found that pairs of annual animal movements have close similar patterns. Pig movements accounted for the largest proportion in each year. Central Thailand was most prominent for the movement of susceptible domestic livestock from 2017 to 2019. For the ERGMs, the microstructures, namely, the edges, mutual, TwoPath, and triangle, were tested and appeared in the annual movement networks. We also found that the higher order microstructures, such as TwoPath and triangle, appeared more than edges and mutual.

Every year, the proportion of animal movement for each species was similar. In each year, upon comparing the movement of each animal species, pigs accounted for the largest proportion of movement, followed by cattle, buffalo, goats, and sheep. Thus, pigs are more likely to influence disease transmission through animal movement than other animals. Therefore, in the case of an outbreak of FMD in Thailand, relevant authorities should first prioritize pig movement control for the monitoring and control of FMD. However, DLD reported that most of the outbreaks of FMD in Thailand from 2017 to 2021 have occurred in cattle [3]. This discrepancy could be attributed to underreporting of pig cases, which was not quantifiable in this study due to lack of case-reporting data. Therefore, our conclusion that pigs may play a major role in transmission is based primarily on movement patterns and should be interpreted with caution. A study based on the 2001 epidemic of FMD in the United Kingdom demonstrated that cattle influenced the nationwide spread of FMD [17]. This is because the United Kingdom has a different animal movement behavior than Thailand. Pigs are the main livestock industry of Thailand, with a high probability of spreading the disease through both local and long-distance spreads. Local spreads can occur in many pig farming sectors, such as backyard or commercial pig movements. Long-distance spreads would be more common in commercial pig movements, while backyard pig movements have a low chance of long-distance spreads. However, control over the movement of other animals in Thailand during an FMD outbreak cannot be ignored.

Based on Table 2 and Figure 2, the ratio of the ties to the actors is high resulting in high centrality values. Considering diseases attributed to animal movements without detection, the spread of FMD is possibly rapid from the source of infection to other provinces owing to the high outdegree and outcloseness. High indegree and high incloseness also indicated that some provinces play an important role as great disease receivers with the fastest incoming disease. Considering betweenness, we also found that the value was high when considering raw scores of betweenness. Similarly, previous studies have identified the superspreaders of FMD using degree and betweenness [18]. Likewise, actors with high values of degree and betweenness play an important role in spreading and receiving disease [19]. We believed that network behavior with high centrality values requires a special and effective active surveillance system to limit the impact of the disease epidemic. Our opinion is consistent with the findings of a study in Switzerland [20]. Figures 1 and 2 show that high animal movement densities were prominent in central Thailand and extended to the northern and northeastern regions. Central Thailand would be a highly suitable area for an active surveillance system. For example, mitigation should be implemented, such as routine, high-frequency FMD testing in pigs and cattle. Moreover, in Australia, a real-time reverse transcription-polymerase chain reaction was used to increase the sensitivity of FMDV in milk samples [21]. Considering Figure 1 (black arrow pointing), the linear directional mean indicated that the movement densities were from east to west in central Thailand. This information would possibly aid relevant authorities in monitoring the direction of the disease and development of animal disease forecasting, which is a part of the early warning system [22]. For example, eastern Thailand is considered a free-zone area for FMD based on forecasting disease direction. Therefore, if FMD is reported in other areas, the relevant authorities need to implement special or additional control measures in this area. Increasing the number of samples to detect FMDV from high-risk animals that move to eastern Thailand could be performed immediately. However, particularly in Figure 1, the kernel density uses the geodesic path to simulate the path, which is not the real route for animal movements. Therefore, we decided not to use the route of animal movements for having too much bias. The driver of animal movement carrier usually goes on a route that does not match the given database. Nevertheless, the origin and destination densities of animal movements are still valid. Thus, resolving the issue may require the application of assistive technologies, such as using Global Positioning System devices for animal movements, which requires further research and planning. Although kernel density used geodesic paths, which may not reflect real transport routes, this was necessary due to the absence of detailed road-network data in the movement dataset. Future studies should consider integrating GPS-based movement tracking or road-network models to reduce geographic bias.

Considering Table 3, animal movements each year had a highly similar pattern based on the network correlation analysis. This is because the movements from/to the farms/sites resulted in small overall changes. Only the number of movements increased over time. Thus, the dynamics of movement patterns would not have changed drastically. In our opinion, designing a database to collect moving data, including using data science, is important for providing data analysis together. This would help the relevant personnel authorities to rapidly interpret the results based on information rather than raw data. This is necessary to monitor the big data of animal movements associated with disease epidemics, such as FMD. As described above, the relevant authorities need to start considering a disease surveillance system using the concept of big data forecasting.

The results of ERGMs indicated that the expression of animal movements initiating from point to point (edges) could be extended to a higher order pattern, such as mutual, TwoPath, and triangle. Thus, the networks would display high complexity. Considering disease occurrence in the networks, complexity networks would be more challenging for limiting the spread of disease. Generally, local occurrence always influences the global effect [15]. Thus, ensuring less complexity in the network, such as by zoning the animal movements, could be one way to limit the magnitude of the spread of the disease. However, it is very difficult to implement a zoning system for animal movements in Thailand in real-world situation owing to the scattered distribution of animal farms throughout the country. Therefore, determining disease-free zones of FMD by DLD located in eastern Thailand is an important policy. This warrants continued attention from the government sectors.

Our study contains certain limitations. First, we assumed equal susceptibility to FMD across all livestock species because the movement dataset did not provide species-specific or serotype-specific infection data. In reality, susceptibility varies; for example, cattle are highly susceptible to all serotypes, while pigs act as amplifying hosts for Serotypes O and Asia-1. This assumption was necessary for modeling but may lead to some oversimplification. Second, we could not obtain simulated higher-order microstructures beyond triangles in the ERGMs due to computational constraints. Future research using advanced computational resources should incorporate these microstructures to better capture complex network dynamics. Third, we could not analyze epidemiological units lower than the provincial level. Nevertheless, an analysis at the provincial level was valuable for understanding the behavior of animal movements in Thailand. Fourth, this level of analysis is consistent with the DLD's disease control framework, where provincial authorities play a major role alongside the governor. We observed that FMD outbreaks often occurred over areas larger than a district or subdistrict, supporting our chosen unit of analysis; however, multilevel analysis could provide deeper insights in future studies. Lastly, we could not incorporate temporal variation or correlate network microstructures with historical FMD outbreak patterns nor validate network-based predictions against actual outbreak data because the dataset lacked time-specific and outbreak location details. Incorporating temporal analysis and validation would strengthen predictive utility and should be explored in future studies.

In conclusion, the results of this study indicate that central Thailand is an important area, and it possibly plays a role in the spread of FMD. Provinces in this area require special attention, particularly in implementing a more active surveillance system. Considering the real situation in Thailand, control measures, such as disease surveys, surveillance at slaughterhouses, laboratory surveillance, farm visit, ordinary movement control, and checkpoints, quarantine, border control, vaccination, case-reported systems, and active case-finding during FMD outbreaks may not be sufficient. The creation of additional effective active surveillance systems is warranted. Moreover, the findings of this study are highly relevant to various regions globally, as they offer valuable insights into the patterns of animal movements and their impact on disease spread, specifically FMD. By understanding the microstructures and dynamics of livestock movements, authorities can enhance their surveillance and control measures, prioritizing high-risk animal movements such as those of pigs. This study underscores the importance of tailored surveillance systems, data-driven decision-making, and the use of advanced technologies such as GPS tracking to monitor and manage animal movements effectively, ultimately aiding in the prevention and control of disease outbreaks on a broader scale.

## 5. Conclusion

This study demonstrated that the movement of domestic livestock susceptible to FMD was very similar each year. The centrality values and mapping presentations indicated the importance of provinces in central Thailand in FMD spreads. ERGMs demonstrated the complexity of animal movements by the appearance of microstructures in the networks, such as edges, mutual, TwoPath, and triangle. Thus, an effective active surveillance system is warranted to limit the spread of FMD. Additionally, this study can be applied to regions with potential FMD spread through animal movements, such as New Zealand and other parts of the world.

## Figures and Tables

**Table 1:** A description of network parameters.

Parameter	Description	Configuration
Outdegree	This parameter evaluated individual actors to determine the number of sources for sending animals to other actors. Raw scores and normalized outdegree centrality were calculated	—
Indegree	This parameter evaluated individual actors to determine the number of sources for obtaining animals to other actors. Raw scores and normalized outdegree centrality were calculated	—
Outcloseness	The shortest path of incoming direction between individual actors and other actors was measured. Raw scores and normalized outdegree centrality were calculated	—
Incloseness	The shortest path of outgoing direction from individual actors to other actors was measured. Raw scores and normalized outdegree centrality were calculated	—
Betweenness	Raw scores and normalized value used to evaluate individual actors determining the actors passing during animal movement. Raw scores and normalized outdegree centrality were calculated	—
Edges	This microstructure shows the relationship of actor pairs with a directional tie. The term is defined as the number of pairs for actor *i* to *j*	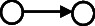
Mutual	This microstructure shows the relationship of the actor pairs with a directional tie. The term is defined as the number of pairs for actor *i* to *j* and *j* to *i*	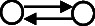
TwoPath	This microstructure shows a pair of edges for actor *i* to *j* and *j* to *k* or actor *i* to *j* and *i* to *k*	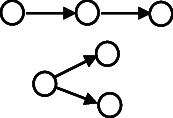
Triangle	This microstructure shows any set of three edges *i* to *j* and *j* to *k* and either *k* to *i* or *i* to *k*	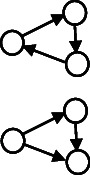

**Table 2:** Descriptive network analysis shows the raw scores and normalized centrality values.

Parameter	Mean (±SD) of the network in the year 2017 (raw score, normalized)	Mean (±SD) of the network in the year 2018 (raw score, normalized)	Mean (±SD) of the network in the year 2019 (raw score, normalized)
Outdegree	4954.80 ± 5846.0, 65.1951 ± 76.9216	5259.80 ± 6448.0, 69.2081 ± 84.8422	5779.10 ± 7047.90, 76.0412 ± 92.7349
Indegree	4955.0 ± 5518.0, 65.1951 ± 72.6011	5260.0 ± 6216.0, 69.2081 ± 81.7879	5779.0 ± 6790.0, 76.0412 ± 89.3371
Outcloseness	106.1430 ± 18.5399, 0.7367 ± 0.1222	116.6490 ± 20.0229, 0.6689 ± 0.1049	115.6360 ± 20.5080, 0.6756 ± 0.1075
Incloseness	106.140 ± 15.3710, 0.7304 ± 0.1021	116.650 ± 18.8210, 0.6677 ± 0.1026	115.640 ± 19.510, 0.6745 ± 0.1051
Betweenness	30.1429 ± 27.6716, 0.0053 ± 0.0049	40.6494 ± 43.3453, 0.0071 ± 0.0076	39.6364 ± 41.2488, 0.0070 ± 0.0072

Abbreviation: SD = standard deviation.

**Table 3:** Network correlation of domestic livestock susceptible to foot and mouth disease (FMD) using a permutation test for the correlation coefficient.

	Year 2017	Year 2018	Year 2019
Year 2017	—	0.979^∗^	0.930^∗^
Year 2018	0.979^∗^	—	0.961^∗^
Year 2019	0.930^∗^	0.961^∗^	—

^∗^ *p* value < 0.01.

**Table 4:** Results of the exponential random graph models (ERGMs) of the networks of domestic livestock susceptible to foot and mouth disease (FMD) from the year 2017 to 2019.

Microstructure	Number of configurations	Estimate	Standard error	*p* value	AIC	GOF
*Year 2017*						
Edges	3546	0.4303	0.0268	< 0.01	7850	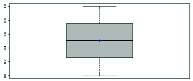
Mutual	1327	0.9116	0.0353	< 0.01	7541	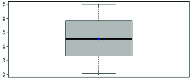
TwoPath	172,768	0.0056	0.0003	< 0.01	7733	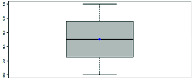
Triangle	172,447	0.0052	0.0002	< 0.01	7467	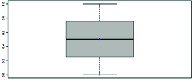

*Year 2018*						
Edges	2871	−0.0376	0.0262	0.15	8113	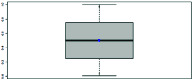
Mutual	1034	0.4973	0.0369	< 0.01	7963	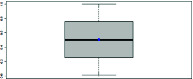
TwoPath	118,194	0.0010	0.0004	< 0.01	8104	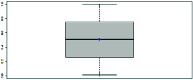
Triangle	112,526	0.0031	0.0002	< 0.01	7976	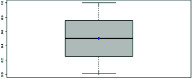

*Year 2019*						
Edges	2971	0.0308	0.0262	0.24	8113	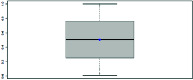
Mutual	1072	0.5375	0.0417	< 0.01	7923	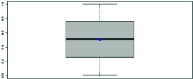
TwoPath	124,975	0.0017	0.0003	< 0.01	8089	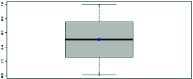
Triangle	121,096	0.0035	0.0002	< 0.01	7922	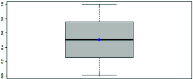

Abbreviations: AIC = Akaike information criterion and GOF = goodness-of-fit.

## Data Availability

Matrix data in the analysis of social networks during this study are included in this published article.

## Nomenclature

FMD: Foot and mouth disease
FMDV: FMD virus
SNA: Social network analysis
ERGMs: Exponential Random Graph Models
GOF: Goodness-of-fit test
SD: Standard deviation

## Supporting Information

Supporting Information 1 Supporting Data 1: The matrix for the movement network in 2017.

Supporting Information 2 Supporting Data 2: The matrix for the movement network in 2018.

Supporting Information 3 Supporting Data 3: The matrix for the movement network in 2019.

Supporting Information 4 Supporting Data 4: Coordinate of provinces.
